# Development and clinical application of a postoperative complication prognosis prediction model for gastric cancer patients based on automated machine learning with body fat rate

**DOI:** 10.3389/fonc.2026.1763139

**Published:** 2026-03-03

**Authors:** Song Xue, Xiangning Dong, Jie Wei, Jiqing Hao

**Affiliations:** 1 Department of Oncology, The First Affiliated Hospital of Anhui Medical University, Hefei, Anhui, China; 2 Department of Oncology, First People’s Hospital of Chuzhou, Chuzhou, Anhui, China

**Keywords:** body fat rate, explainable artificial intelligence (XAI), gastric cancer, improved hike optimization algorithm, machine learning, postoperative complications

## Abstract

**Objective:**

To develop an automated machine learning (AutoML) framework integrating body composition indices—notably Body Fat Rate (BFR)—and clinicopathological features for predicting postoperative complications in gastric cancer patients, addressing limitations of traditional body mass index (BMI) assessment and enhancing clinical translatability.

**Methods:**

In this retrospective cohort study, 1,023 gastric cancer patients undergoing radical gastrectomy (January 2020–January 2025) were enrolled across two hospitals (716 training, 307 testing). A dual-optimization workflow included: (1) Simultaneous feature selection and hyperparameter tuning via the Improved Hike Optimization Algorithm (IHOA); (2) Class imbalance mitigation using synthetic minority oversampling technique (SMOTE). Model performance was evaluated through accuracy, sensitivity, specificity, F1-score, area under the receiver operating characteristic curve (AUC-ROC), area under the precision-recall curve (AUC-PR), calibration curves, and decision curve analysis (DCA). Feature robustness was validated using least absolute shrinkage and selection operator regression, while SHapley Additive exPlanations (SHAP) interpreted predictor contributions. A MATLAB-based proof-of-concept prototype visualization tool was developed for implementation.

**Results:**

In independent testing, AutoML maintained robust performance (ROC-AUC = 0.9380, PR-AUC = 0.9262). DCA revealed greater net clinical benefit across risk thresholds (1%–93%) compared to conventional methods, with sustained high-level stability confirming superior generalizability. Calibration curves demonstrated optimal probabilistic prediction (lowest test-set Brier score = 0.111). SHAP analysis identified BFR, visceral fat density (VFD), visceral fat area (VFA), skeletal muscle area (SMA), C-reactive protein (CRP), BMI, Age and lymphadenectomy extent as key predictors.

**Conclusion:**

The AutoML prediction model developed in this study achieves both high precision and strong interpretability. Its visualized tool effectively overcomes barriers to clinical translation, providing intelligent decision support for early warning and personalized intervention of postoperative complications in gastric cancer.

## 1 Introduction

Gastric cancer, a highly prevalent malignant tumor of the digestive system globally, exhibits both incidence and mortality rates ranking among the highest for malignancies in China, posing a severe threat to population well-being (1). Surgical resection serves as the primary curative approach for gastric cancer; however, the risk of postoperative recurrence and metastasis remains elevated, with a 5-year survival rate of merely 30%–40% and significant interindividual prognostic heterogeneity (2). Thus, accurate postoperative complication risk assessment and personalized treatment strategies are crucial for improving patient survival outcomes. Traditional prognostic evaluation systems predominantly rely on pathological indicators such as tumor stage and differentiation grade. While clinically valuable to some extent, these parameters fail to comprehensively reflect patients’ systemic status and disease progression potential, limiting assessment precision (3).

Advancing precision medicine has propelled multidimensional prognostic prediction models to the forefront of oncology research. Body Fat Rate (BFR), a vital indicator reflecting nutritional status and metabolic levels, has garnered widespread attention for its association with cancer prognosis. Studies confirm that abnormal BFR levels (either elevated or reduced) may heighten postoperative recurrence risk and shorten survival in gastric cancer patients by impairing immune function, exacerbating inflammatory responses, and altering tumor microenvironments (4). Notably, the traditional Body Mass Index (BMI), employed for obesity assessment, inadequately captures adipose tissue accumulation patterns and spatial distribution (5). In contrast, BFR measurement offers rapid evaluation with operational simplicity, low cost, and zero radiation exposure risk—making it suitable for large-scale population screening. The incorporation of BFR represents a significant advance beyond conventional anthropometric indicators such as BMI. Unlike BMI, which quantifies general body mass without distinguishing adipose distribution or metabolic status, BFR specifically evaluates fat deposition patterns that directly modulate inflammatory cascades in surgical contexts. This physiological distinction provides a novel dimension for risk stratification previously unattainable through traditional nutritional assessments. Nevertheless, whether BFR serves as an independent predictor of postoperative complication risk in gastric cancer requires further investigation (6).

The emergence of Automated Machine Learning (AutoML) technology provides innovative solutions for mining and modeling complex medical data. Compared to conventional machine learning, AutoML automates feature engineering, model selection, and hyperparameter tuning—reducing subjectivity and operational complexity while effectively uncovering latent data correlations to significantly enhance model performance and generalizability (7). Currently, AutoML has demonstrated promising applications in prognostic prediction for cancers such as lung and breast cancer, yet research integrating AutoML with BFR for postoperative complication risk assessment in gastric cancer remains scarce.

To address this gap, this study aims to integrate BFR, clinicopathological features, and laboratory test results of gastric cancer patients to construct a postoperative complication risk prediction model using AutoML technology. Through systematic validation of the model’s predictive accuracy, stability, and clinical utility, we seek to elucidate the prognostic value of BFR in gastric cancer and provide novel insights for precision prevention and clinical management.

## 2 Methods

### 2.1 Study participants and data collection

This retrospective cohort study enrolled 1,023 gastric cancer patients who underwent radical gastrectomy at The First Affiliated Hospital of Anhui Medical University (n=815) and Chuzhou First People’s Hospital (n=208) from January 2020 to January 2025. Ethical approval (No. 83220465) was granted by the Institutional Review Board of The First Affiliated Hospital of Anhui Medical University, with waived informed consent in accordance with the Declaration of Helsinki. Patients were selected based on strict criteria:

Inclusion criteria: (1) Histopathologically confirmed non-metastatic gastric adenocarcinoma; (2) Radical gastrectomy performed; (3) No neoadjuvant radiotherapy or chemotherapy; (4) Complete clinical and follow-up records.

Exclusion criteria: (1) Non-gastric malignancy or distant metastasis; (2) Incomplete body composition data [BFR, BMI, Visceral Fat Density (VFD), Visceral Fat Area (VFA)]; (3) Concurrent malignancies.

Data were extracted from electronic medical records using structured templates and verified by two certified researchers. Collected features included: (1) Demographics: Age, sex. (2) Clinicopathological characteristics: AJCC 8th edition TNM stage (8), tumor location (cardia/body/antrum), histological grade (well-moderate/poor-undifferentiated), surgical approach (laparoscopic/open), lymphadenectomy extent (D1/D2). (3) Body composition metrics: BMI (kg/m²) calculated from preoperative height/weight. VFD (g/cm³), BFR (%), VFA (cm²), Subcutaneous Fat Area (SFA, cm²), and Skeletal Muscle Area (SMA, cm²) derived from preoperative abdominal CT scans at L3 level using Slice-O-Matic software. (4) Preoperative laboratory markers (within 1 week pre-surgery): Serum albumin (ALB, g/L), prealbumin (PA, mg/L), hemoglobin (Hb, g/L), white blood cell count (WBC, ×10^9^/L), C-reactive protein (CRP, mg/L). (5) Outcome: Postoperative complications within 30 days (Clavien-Dindo grade ≥II defining clinically significant events) (9).

This study minimized data loss at the source through stringent inclusion and exclusion criteria. In the final analytical sample, only a few variables exhibited minimal random missing values (all missing rates ≤6.0%). To ensure sample completeness and statistical power for subsequent analyzes and predictive models, different single imputation strategies were adopted based on variable types and distribution characteristics: mean imputation was used for normally distributed continuous variables (e.g., SMA, ALB, Hb), while median imputation was applied to non-normally distributed continuous variables (e.g., BFR, VFA, VFD, WBC, CRP). All categorical variables were complete in this dataset; however, planned mode imputation would address minor missingness if present, as detailed in **Table 1**.

**Table 1:** Data missingness and handling methods for study variables.

Feature	Missing count (Missing rate)	Handling method
Categorical variables		
Sex	0 (0%)	No missing
TNM stage	0 (0%)	No missing
Tumor location	0 (0%)	No missing
Histological differentiation	0 (0%)	No missing
Surgical approach	0 (0%)	No missing
Lymphadenectomy	0 (0%)	No missing
Continuous variables (Normally distributed, Mean ± SD)		
Age	0 (0%)	No missing
BMI	0 (0%)	No missing
SMA	8 (3.2%)	Mean imputation
ALB	5 (2.0%)	Mean imputation
Hb	3 (1.2%)	Mean imputation
Continuous variables (Non-normally distributed, Median [Q1, Q3])		
BFR	12 (4.8%)	Median imputation
VFA	12 (4.8%)	Median imputation
SFA	12 (4.8%)	Median imputation
VFD	15 (6.0%)	Median imputation
PA	5 (2.0%)	Median imputation
WBC	10 (4.0%)	Median imputation
CRP	10 (4.0%)	Median imputation

### 2.2 Automated machine learning

815 patients from The First Affiliated Hospital of Anhui Medical University served as the training set, while 208 patients from Chuzhou First People’s Hospital constituted the testing set. All models were implemented in MATLAB 2024b. Five-fold cross-validation was applied to the training set to mitigate overfitting, and Synthetic Minority Over-sampling Technique (SMOTE) was executed on the training set to address class imbalance.

We propose an AutoML framework driven by the Improved Hike Optimization Algorithm (IHOA) to jointly optimize feature selection and hyperparameter tuning for predicting postoperative complications. The original Hike Optimization Algorithm (HOA) is a novel metaheuristic algorithm inspired by natural aurora phenomena. Key improvements were implemented: (1) Population initialization using Logistic chaotic mapping, mathematically defined as $x_{n+1}=\mu x_{n}(1-x_{n})$ (where μ∈(3.57,4] denotes the chaotic parameter), which significantly enhances initial traversal in the solution space and facilitates escaping local optima; (2) A dynamic Lévy flight strategy to adjust search step sizes, computed as step=α⊕Le'vy(β) (where α is the scale parameter, ⊕ represents the dot product, and Le'vy(β) follows a Lévy distribution with exponent β). This strategy employs larger steps for global exploration in early iterations and adaptively reduces step sizes for local exploitation in later stages, achieving dynamic exploration-exploitation balance. The enhanced IHOA was first evaluated on the CEC2022 benchmark test functions to validate its superiority over the original HOA and other mainstream metaheuristic algorithms. At the empirical level, dual validation was performed through clinical prediction models: robustness testing via artificial injection of progressive data perturbations (0%–15% noise and 0%–30% missing values), alongside a feature selection module assessing model complexity control capabilities.

**Figure 1:**
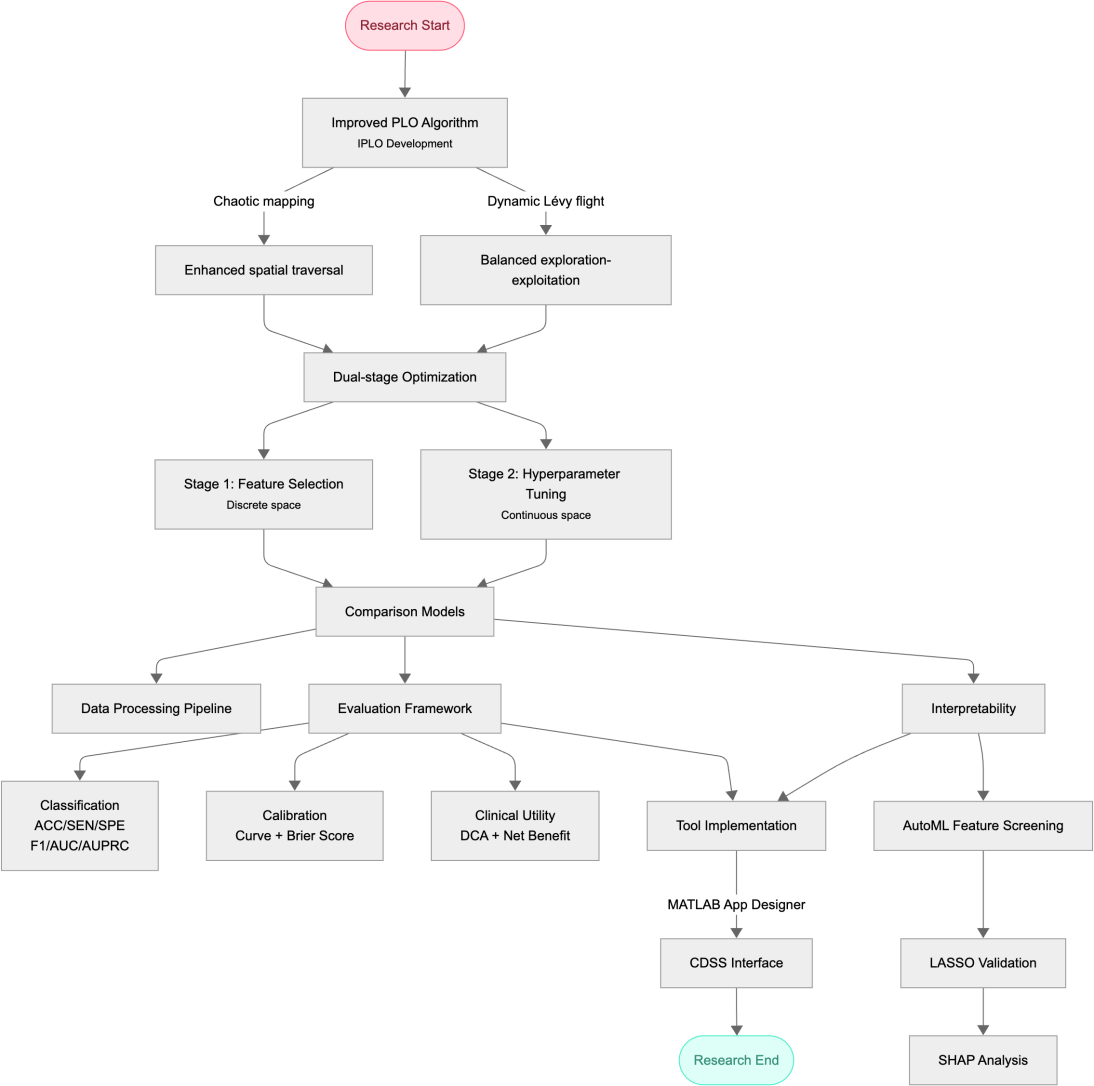
Technical workflow diagram.

The core of this AutoML framework is a two-stage optimization workflow (pseudocode in **Appendix A**). In Stage 1 (discrete space optimization), IHOA is applied to feature selection, where each candidate solution (i.e., individual position) is encoded as a binary vector representing a feature subset. Model-based feature importance weights (e.g., XGBoost) serve as prior knowledge to guide the IHOA search, with the fitness function being a weighted combination of performance metrics (e.g., AUC) evaluated on the validation set using a base classifier (e.g., logistic regression). This selects high-weight feature subsets with strong discriminative power and low redundancy. In Stage 2 (continuous space optimization), IHOA optimizes hyperparameters of the target prediction model—integrating classical algorithms including support vector machines, random forests, and gradient boosting trees—for the optimal subset identified in Stage 1. Hyperparameter combinations are encoded as continuous vectors to maximize prediction performance on the validation set. Finally, for comprehensive evaluation, the proposed framework was compared against six widely-used machine learning models: Logistic Regression (LR), Support Vector Machine (SVM), Adaptive Boosting (AdaBoost), Extreme Gradient Boosting (XGBoost), and LightGBM. All models were evaluated using identical training-testing datasets to ensure fairness. The technical workflow is shown in **Figure 1**.

### 2.3 Evaluation metrics

A multidimensional evaluation system was established: (1) Classification performance: For the predictive model, six core metrics were adopted to systematically assess discriminative ability and stability in class-imbalanced scenarios: Accuracy (ACC), Sensitivity (SEN), Specificity (SPE), Precision (PRE), F1 score (harmonic mean of precision and recall), Area Under the ROC Curve (ROC-AUC), and Area Under the Precision-Recall Curve (PR-AUC). (2) Calibration performance: Calibration curves combined with the Brier score (lower scores indicate higher prediction accuracy) were used to evaluate probability prediction precision; the Hosmer–Lemeshow test (χ² statistic) and Spiegelhalter test (z statistic) quantified calibration deviation (P > 0.05 indicated good calibration). (3) Clinical application: Decision Curve Analysis (DCA) was applied to quantify clinical utility by calculating the net benefit (NB) at different threshold probabilities:


$$NB=\frac{TP}{N}-\frac{FP}{N}\times \frac{p_{t}}{1-p_{t}}$$

where *TP* is true positives, *FP* is false positives, *N* is total sample size, and pt is the risk threshold. By comparing *NB* with reference lines for traditional intervention strategies, the effective interval for model-assisted decision-making was validated.

### 2.4 Interpretability analysis

Post AutoML feature screening, robustness was verified via LASSO regression, followed by SHapley Additive exPlanations (SHAP) for clinical interpretability: AutoML feature screening: Automatically identified prognosis-associated features. LASSO regression: Validated feature sparsity and stability using regularization. SHAP analysis: By calculating Shapley values for each feature, quantitative attribution of feature contributions to individual predictions was achieved; summary plots were comprehensively used to display global feature importance rankings, while waterfall plots, decision path plots, and force plots were employed to visually illustrate the interpretation process of specific prediction cases, thereby systematically revealing the model’s intrinsic decision-making logic.

### 2.5 Interactive tool development

A proof-of-concept clinical decision support tool was developed using MATLAB 2024a App Designer due to its accelerated prototyping workflow for model-visualization integration and seamless compatibility with existing statistical preprocessing pipelines (Statistical Package for the Social Sciences, SPSS), integrating the optimal machine learning model.

### 2.6 Statistical analyzes

All study data were uniformly imported into the SPSS 26.0 statistical analysis platform for standardized processing. Continuous variables with normal distribution were expressed as mean ± standard deviation (x̄± s), while those with non-normal distribution were denoted as median (interquartile range) (M [IQR]); categorical variables were expressed as frequency and percentage (n(%)). For inter-group comparisons, continuous variables first underwent normality testing: if both groups conformed to normal distribution, univariate t-test was applied; otherwise, Mann-Whitney U test was used. Categorical variables were compared using Pearson’s chi-square test. Statistical significance was determined based on p-values (two-tailed test, significance threshold α=0.05), and results were presented in tabular formats.

## 3 Results

### 3.1 Characteristics of study participants

A total of 1,023 subjects were included, with a mean age of 62.65 ± 10.16 years. Among them, 686 (67.06%) were male and 337 (32.94%) were female. The comparison of general characteristics between the training set and testing set is presented in **Table 2**. The results demonstrate balanced distributions across most demographic and clinical baseline features (P > 0.05), with statistically significant differences observed only in isolated variables. Such limited discrepancies following random partitioning more closely reflect the natural variability inherent in real-world data, avoiding idealization biases that may arise from artificial overmatching. This consequently enhances the extrapolation potential and reliability of the models developed in this study for broader clinical scenarios.

**Table 2:** Baseline characteristics of enrolled participants.

Features	Training set (n=815)	Testing set (n=208)	Statistic	P-value
Postoperative complications, n (%)			χ²=1.326	0.250
No	689 (84.54)	169 (81.25)		
Yes	126 (15.46)	39 (18.75)		
Age (years), Mean ± SD	62.54 ± 10.15	63.07 ± 10.21	t=-0.873	0.383
Sex, n(%)			χ²=1.161	0.281
Female	275 (33.74)	62 (29.81)		
Male	540 (66.26)	146 (70.19)		
TNM stage, n(%)			χ²=2.466	0.482
I	238 (29.20)	72 (34.62)		
II	250 (30.67)	60 (28.85)		
III	281 (34.48)	64 (30.77)		
IV	46 (5.64)	12 (5.77)		
Tumor location, n(%)			χ²=2.734	0.255
Cardia	170 (20.86)	53 (25.48)		
Gastric antrum	369 (45.28)	83 (39.90)		
Gastric body	276 (33.87)	72 (34.62)		
Histological differentiation, n(%)			χ²=2.264	0.519
Moderately differentiated	264 (32.39)	68 (32.69)		
Poorly differentiated	366 (44.91)	87 (41.83)		
Undifferentiated	93 (11.41)	22 (10.58)		
Well-differentiated	92 (11.29)	31 (14.90)		
Surgical approach, n(%)			χ²=0.208	0.649
Laparoscopic	523 (64.17)	137 (65.87)		
Open Surgery	292 (35.83)	71 (34.13)		
Lymphadenectomy, n(%)			χ²=11.847	<0.001
D1	141 (17.30)	58 (27.88)		
D2	674 (82.70)	150 (72.12)		
BMI (kg/m²), Mean ± SD	22.82 ± 3.45	22.88 ± 3.10	t=-0.276	0.782
BFR (%), M [Q1, Q3]	28.6 [24.2, 33.0]	28.6 [24.3, 33.6]	Z=-0.294	0.769
VFA (cm²), M [Q1, Q3]	100.7 [64.2, 130.6]	106.7 [76.7, 147.6]	Z=-2.809	0.005
SFA (cm²), M [Q1, Q3]	121.4 [87.7, 154.0]	121.0 [86.5, 156.0]	Z=0.208	0.836
SMA (cm²), Mean ± SD	119.04 ± 25.95	118.40 ± 24.13	t=0.338	0.735
VFD (g/cm³), M [Q1, Q3]	0.84 [0.77, 0.91]	0.84 [0.78, 0.92]	Z=-0.336	0.737
ALB (g/L), Mean ± SD	38.47 ± 5.33	38.48 ± 5.74	t=-0.036	0.971
PA (mg/L), M [Q1, Q3]	209.0 [176.5, 245.0]	217.0 [175.8, 253.0]	Z=-0.327	0.744
Hb (g/L), Mean ± SD	124.22 ± 18.20	125.23 ± 18.19	t=-0.716	0.474
WBC (×109/L), M [Q1, Q3]	6.4 [5.1, 7.9]	6.5 [5.2, 7.7]	Z=0.358	0.720
CRP (mg/L), M [Q1, Q3]	5.0 [0.6, 9.6]	7.3 [1.5, 11.7]	Z=-3.011	0.002

### 3.2 Comparative analysis of improved swarm intelligence algorithm performance

**Figure 2:**
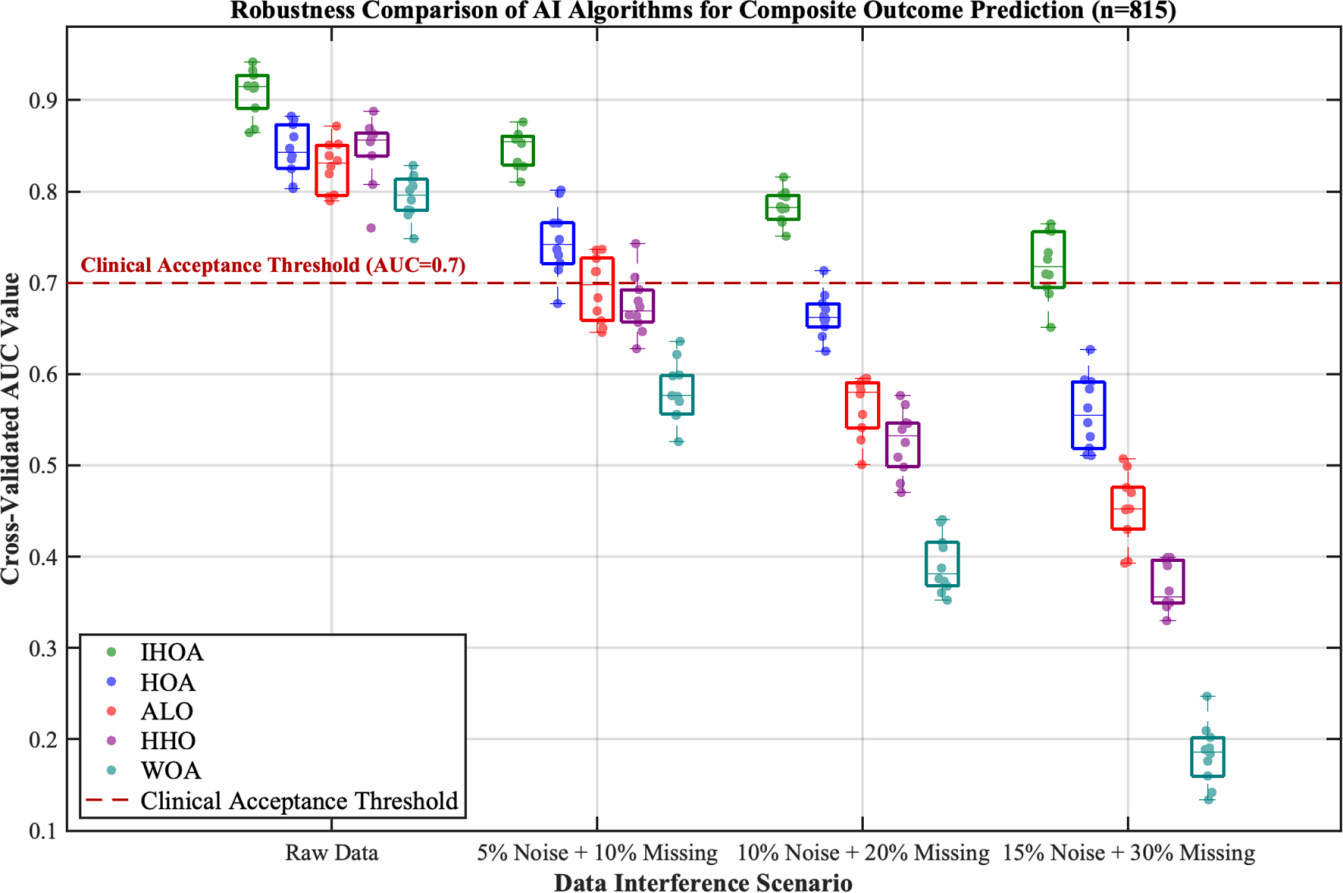
Robustness comparison of intelligent algorithms in predicting composite postoperative complication risk.

The IHOA demonstrated significant optimization advantages through standardized testing (see **Appendix B**). By artificially injecting progressive data perturbations, this study evaluated the performance of the prognosis prediction model based on real-world clinical data. Experimental results revealed that all intelligent algorithms exhibited declining performance trends as perturbation intensity increased (**Figure 2**). Under original data conditions, the five algorithms achieved an average prediction accuracy of AUC = 0.84; however, under extreme perturbations (15% noise + 30% missing values), model performance substantially decreased to an average AUC = 0.46—a relative decline of 45.4%. Critically, the IHOA algorithm exhibited optimal stability: its prediction accuracy decreased from AUC = 0.91 under original conditions to AUC = 0.73 in high-perturbation scenarios, with a relative decrease rate (19.7%) markedly lower than other algorithm groups (all >36%).

**Figure 3:**
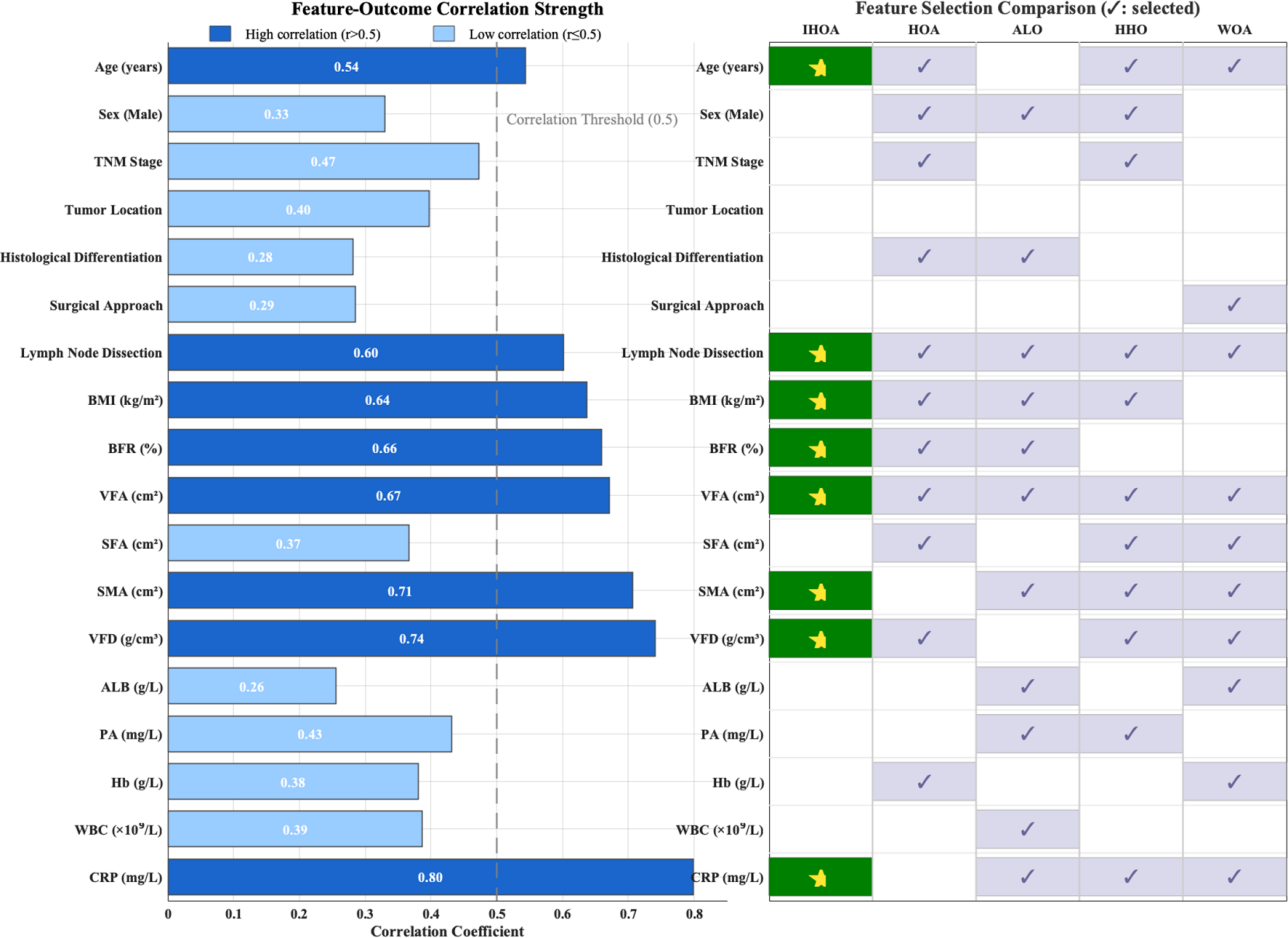
Clinical feature analysis and feature selection optimization.

Note: This figure demonstrates the stability performance of five intelligent algorithms (IHOA/HOA/ALO/HHO/WOA) under increasing data interference scenarios. The boxplots represent the distribution of cross-validated AUC values from 10 repeated experiments, while the scatter points indicate single experimental results. Algorithm color coding: IHOA (forest green), HOA (blue), ALO (red), HHO (purple), WOA (cyan). The red dashed line indicates the clinically acceptable threshold (AUC = 0.7), values below which are considered insufficient for clinical reference.

Note: This study compared the correlation strength between 18 clinical features and postoperative complication risk, and evaluated the feature selection performance of the IHOA algorithm against other optimization algorithms (HOA, ALO, HHO, WOA). The left panel displays the correlation coefficients (r) between each feature and the outcomes, with dark blue indicating highly correlated features (r>0.5), light blue indicating weakly correlated features (r ≤ 0.5), and the gray dashed line representing the correlation threshold of 0.5. The right panel shows the feature selection results of the algorithms, where dark green squares represent features selected by IHSO, and light blue squares represent features selected by other algorithms.

At the key feature identification level, the IHOA algorithm demonstrated superior concise characteristics (**Figure 3**). It consistently screened 8 highly correlated predictors (average correlation coefficient r=0.67, SD = 0.08)—a 26% reduction compared to the average 10.8 features selected by other algorithms. This feature refinement mechanism effectively reduced model complexity while maintaining predictive accuracy.

### 3.3 Model training outcomes

**Figure 4:**
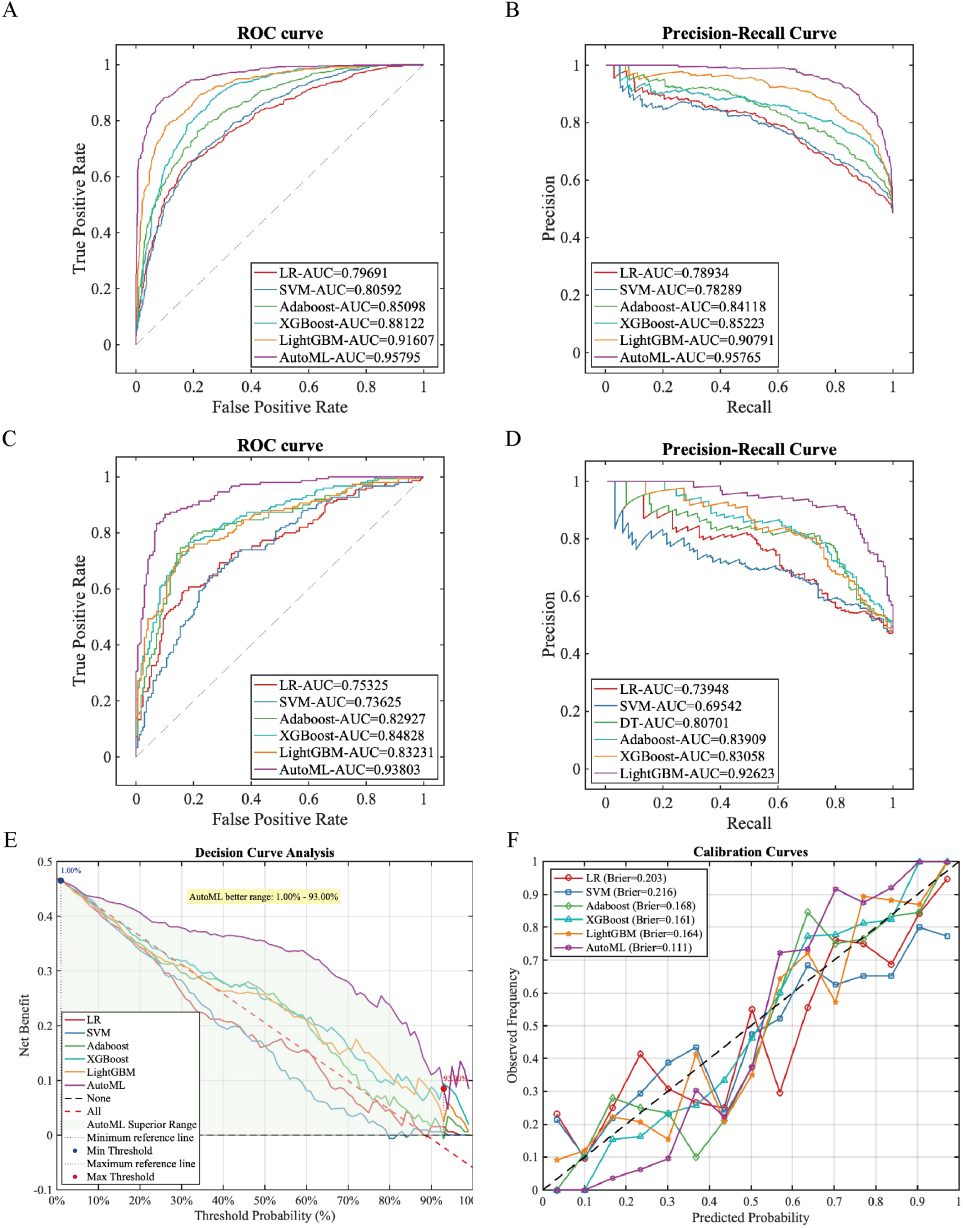
Training and validation performance of prediction models. **(A)** Training ROC curve; **(B)** Training PR curve; **(C)** Test ROC curve; **(D)** Test PR curve; **(E)** Test DCA curve; **(F)** Test calibration curve.

Six machine learning models were systematically evaluated on the training set for accuracy, sensitivity, specificity, F1-score, and area-under-curve metrics. The AutoML model demonstrated comprehensive superiority, achieving ROC-AUC of 0.9580 and PR-AUC of 0.9577 (**Table 3**; **Figures 4A, B**). Notably, AutoML exhibited exceptional F1-score (0.8855), indicating strong clinical utility in balancing precision-recall tradeoffs. The algorithm ultimately selected eight key features: BFR, VFA, VFD, SMA, CRP, lymphadenectomy extent, BMI and Age.

**Table 3:** Performance metrics of prediction models.

Data set	Models	PRE	SEN	SPE	ACC	F1	ROC-AUC (95% CI)	PR-AUC (95% CI)
Training set	LR	0.7743	0.6492	0.8215	0.7379	0.7063	0.7969 [0.785, 0.809]	0.7893 [0.777, 0.802]
	SVM	0.6899	0.7908	0.6647	0.7259	0.7369	0.8059 [0.793, 0.818]	0.7829 [0.769, 0.797]
	Adaboost	0.7714	0.7631	0.7866	0.7752	0.7672	0.8510 [0.839, 0.863]	0.8412 [0.829, 0.854]
	XGBoost	0.7601	0.8723	0.7402	0.8043	0.8123	0.8812 [0.870, 0.892]	0.8522 [0.840, 0.865]
	LightGBM	0.8787	0.7800	0.8984	0.8409	0.8264	0.9161 [0.907, 0.925]	0.9079 [0.898, 0.918]
	AutoML	0.9161	0.8569	0.9260	0.8925	0.8855	0.9580 [0.951, 0.965]	0.9577 [0.950, 0.965]
Testing set	LR	0.6606	0.7267	0.6686	0.6959	0.6921	0.7533 [0.725, 0.781]	0.7395 [0.711, 0.768]
	SVM	0.6471	0.7333	0.6450	0.6865	0.6875	0.7363 [0.708, 0.765]	0.6954 [0.666, 0.725]
	Adaboost	0.8116	0.7467	0.8462	0.7994	0.7778	0.8293 [0.802, 0.857]	0.8070 [0.779, 0.835]
	XGBoost	0.7877	0.7667	0.8166	0.7931	0.7770	0.8483 [0.822, 0.875]	0.8391 [0.812, 0.866]
	LightGBM	0.8029	0.7333	0.8402	0.7900	0.7666	0.8323 [0.805, 0.860]	0.8306 [0.803, 0.858]
	AutoML	0.9124	0.8333	0.9290	0.8840	0.8711	0.9380 [0.917, 0.959]	0.9262 [0.904, 0.948]

In independent testing, AutoML maintained robust performance (ROC-AUC = 0.9380, PR-AUC = 0.9262; **Figures 4C, D**). Decision curve analysis (**Figure 4E**) revealed greater net clinical benefit across risk thresholds (1%–93%) compared to conventional methods, with sustained high-level stability confirming superior generalizability. Calibration curves (**Figure 4B**) demonstrated optimal probabilistic prediction (lowest test-set Brier score = 0.111).

Calibration statistics for all models are presented in **Table 4**. The Brier score demonstrated that the AutoML model (0.111) achieved the highest prediction accuracy, significantly outperforming other models (reduction of approximately 32–49%). Calibration intercept analysis revealed that all models exhibited a mild overestimation tendency (intercepts ranged from –0.151 to –0.052). The AutoML’s calibration intercept was closest to the ideal value of 0 (–0.052), with minimal calibration bias, demonstrating significant superiority over other models. Calibration slope analysis indicated that AutoML’s slope was closest to the ideal value of 1 (1.082), confirming its optimal balance between discrimination and calibration.

**Table 4:** Calibration curve statistical results.

Model	Brier score	Calibration intercept	Calibration slope	Hosmer-Lemeshow χ²	Hosmer-Lemeshow p-value	Spiegelhalter z	Spiegelhalter p-value
LR	0.203	-0.132	0.689	19.110	0.014	-1.010	0.311
SVM	0.216	-0.139	0.565	29.850	0.000	-1.180	0.239
Adaboost	0.168	-0.151	1.038	20.850	0.008	-1.100	0.269
XGBoost	0.161	-0.090	1.480	11.400	0.180	-0.860	0.388
LightGBM	0.164	-0.095	1.027	10.820	0.212	-0.710	0.478
AutoML	0.111	-0.052	1.082	8.450	0.391	-0.450	0.653

The Hosmer-Lemeshow test showed that AutoML (χ²=8.450, p=0.391) passed the test with the highest p-value, indicating optimal calibration goodness-of-fit; LightGBM (χ²=10.820, p=0.212) and XGBoost (χ²=11.400, p=0.180) also passed the test, suggesting good calibration, while other models exhibited calibration bias (p<0.05). Collectively, across all calibration metrics, the AutoML model demonstrated optimal performance in every dimension, validating its excellence in both prediction accuracy and calibration capability.

### 3.4 Key predictor analysis

#### 3.4.1 LASSO regression

**Figure 5:**
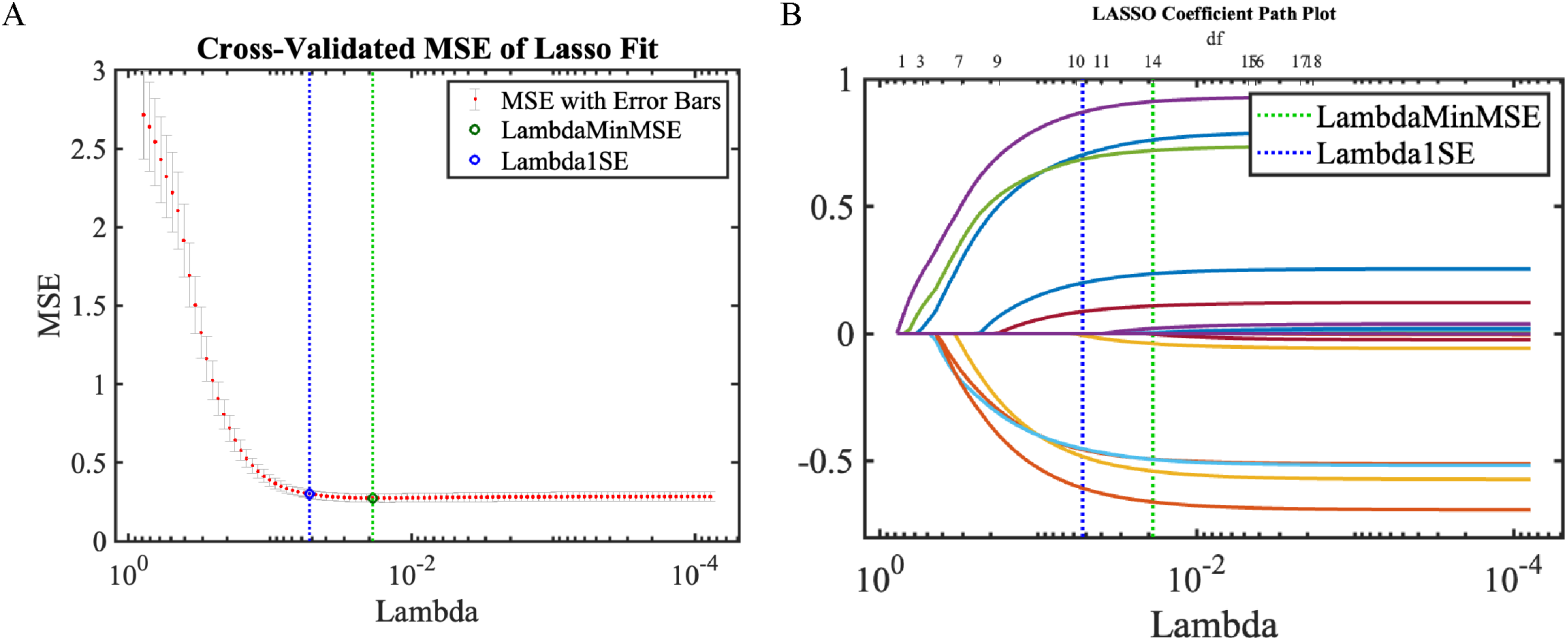
LASSO regression results. **(A)** Coefficient trajectories; **(B)** Cross-validation error plot.

LASSO regression identified 10 predictors within one standard error of minimum MSE (Lambda1SE): BFR, VFA, VFD, SMA, CRP, lymphadenectomy extent, BMI, ALB, SFA, and age, fully encompassing the AutoML-selected features (**Figure 5**).

#### 3.4.2 SHAP analysis

**Figure 6:**
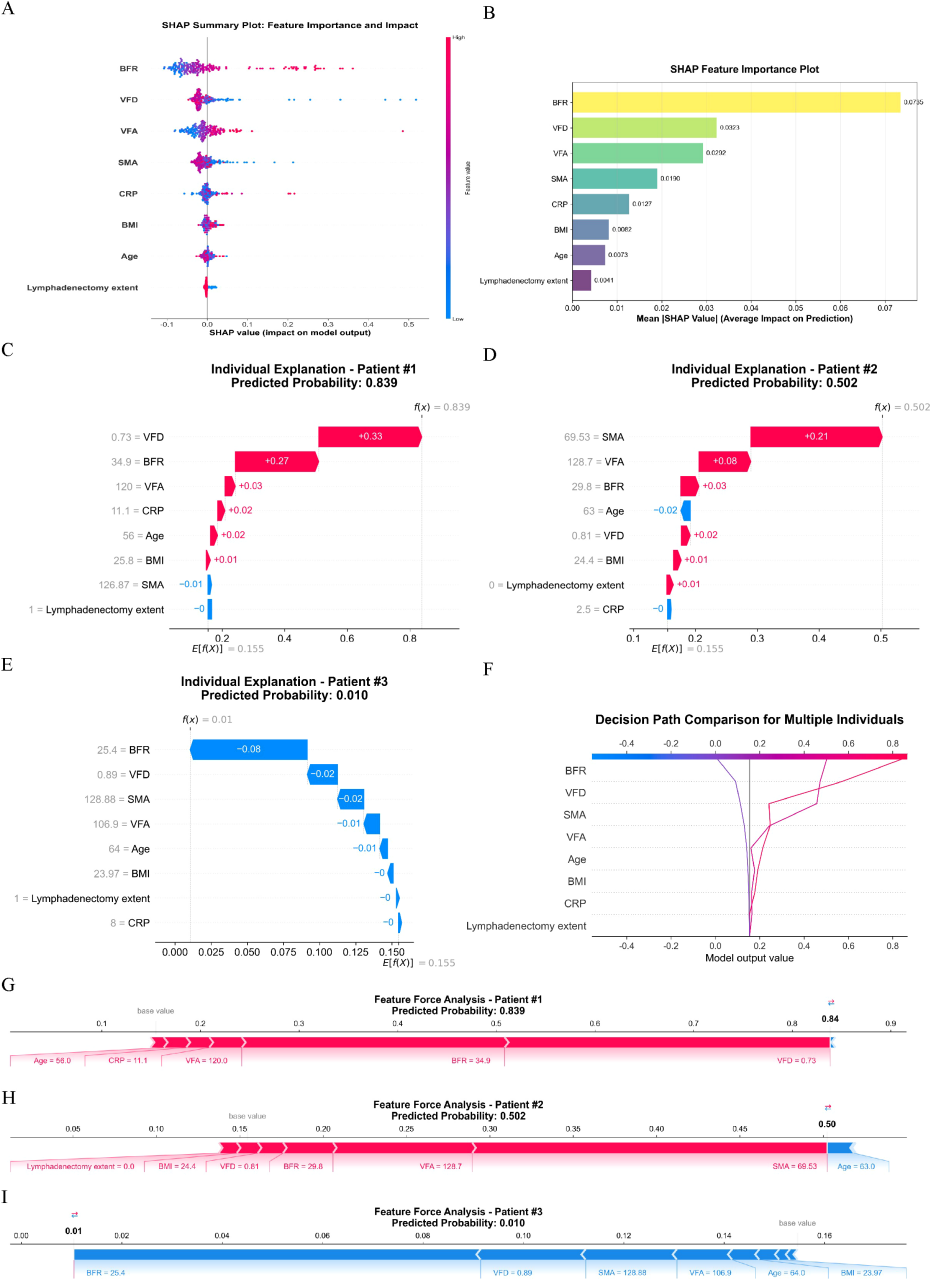
SHAP Interpretability Analysis. **(A)** The Shapley summary plot; **(B)** The Shapley feature importance plot; **(C–E)** Waterfall plots illustrate the cumulative contribution process of each feature to individual patient predictions. The baseline value represents the model’s average prediction for all patients, while feature contributions show how each feature affects the final prediction (red indicating increased risk, blue indicating decreased risk). The sum of all feature contributions yields the final predicted value; **(F)** The decision path plot compares decision pathways across multiple patients, demonstrating how different feature combinations lead to varying prediction outcomes. The horizontal axis shows predicted probabilities, the vertical axis lists features, and the curved pathways trace decision routes from baseline values to final predictions; **(G–I)** Force plots visually demonstrate how each feature “pushes” predictions toward higher or lower risk directions. Red arrows indicate features pushing predictions toward higher risk, blue arrows indicate features pushing toward lower risk, with arrow length representing the magnitude of influence.

Feature importance ranking determined via SHAP analysis was: BFR > VFD > VFA > SMA > CRP > BMI > Age > lymphadenectomy extent (**Figures 6A, B**). Analysis of decision paths across different risk levels (**Figure 6F**) reveals systematic differences in feature combinations between high-risk and low-risk patients. High-risk patients exhibit distinct rightward path deviations, indicating synergistic effects of multiple risk factors. Crucially, the interpretability analysis identified BFR as the foremost predictor through rigorous SHAP ranking, where optimal cutoff values synergistically interacted with visceral fat metrics to stratify complication risks. This multidimensional integration of body composition parameters enabled unprecedented granularity in predicting postoperative complication risk, with BFR contributing significantly to the model’s discriminative ability.

Case Interpretation.

Case 1: High-risk patient (ID 1, predicted probability: 83.85%).

This 56-year-old male had BFR = 34.9%, VFA = 120.0 cm², VFD = 0.73 g/cm³, SMA = 126.87 cm², CRP = 11.1 mg/L, underwent D2 lymphadenectomy, and BMI = 25.8 kg/m². SHAP analysis identified two dominant risk drivers: VFD (SHAP = 0.3302) and BFR (SHAP = 0.2665). Suboptimal VFD (0.73 g/cm³ < median 0.84 g/cm³) suggesting inflammatory status was the primary risk amplifier, compounded by elevated BFR (34.9% > median 28.6%). Moderate contributions came from VFA (SHAP = 0.0329) and CRP (SHAP = 0.0234). SMA’s minor protective effect (SHAP=–0.0052) was insufficient to counterbalance overall risk.

Case 2: Moderate-risk patient (ID 2, predicted probability: 50.23%).

This 63-year-old male had BFR = 29.8%, VFA = 128.7 cm², VFD = 0.81 g/cm³, SMA = 69.53 cm², CRP = 2.5 mg/L, underwent D1 lymphadenectomy, and BMI = 24.4 kg/m². Severely reduced SMA (69.53 cm² < mean 119.04 cm²) drove risk (SHAP = 0.2129), amplified by elevated VFA (128.7 cm² > median 100.7 cm²; SHAP = 0.0835). Typical BFR (29.8% ≈ median 28.6%) and VFD (0.81 g/cm³ ≈ median 0.84 g/cm³) contributed minimally (SHAP = 0.0300/0.0151). D1 lymphadenectomy slightly increased risk (SHAP = 0.0108), while protective age (SHAP=–0.0160) and CRP (SHAP=–0.0012) partially offset hazards.

Case 3: Low-risk patient (ID 3, predicted probability: 1.04%).

This 64-year-old male had BFR = 25.4%, VFA = 106.9 cm², VFD = 0.89 g/cm³, SMA = 128.88 cm², CRP = 8.0 mg/L, underwent D2 lymphadenectomy, and BMI = 23.97 kg/m². Synergistic protective effects were observed: reduced BFR (25.4% < median 28.6%; SHAP=–0.0810), optimal VFD (0.89 g/cm³ > median 0.84 g/cm³; SHAP=–0.0209), and SMA near mean (SHAP=–0.0182). Elevated CRP (8.0 mg/L) was counteracted by dominant protective features. This case exemplifies the model’s ability to identify low-risk profiles when multiple favorable factors coexist.

### 3.5 Interactive clinical tool

**Figure 7:**
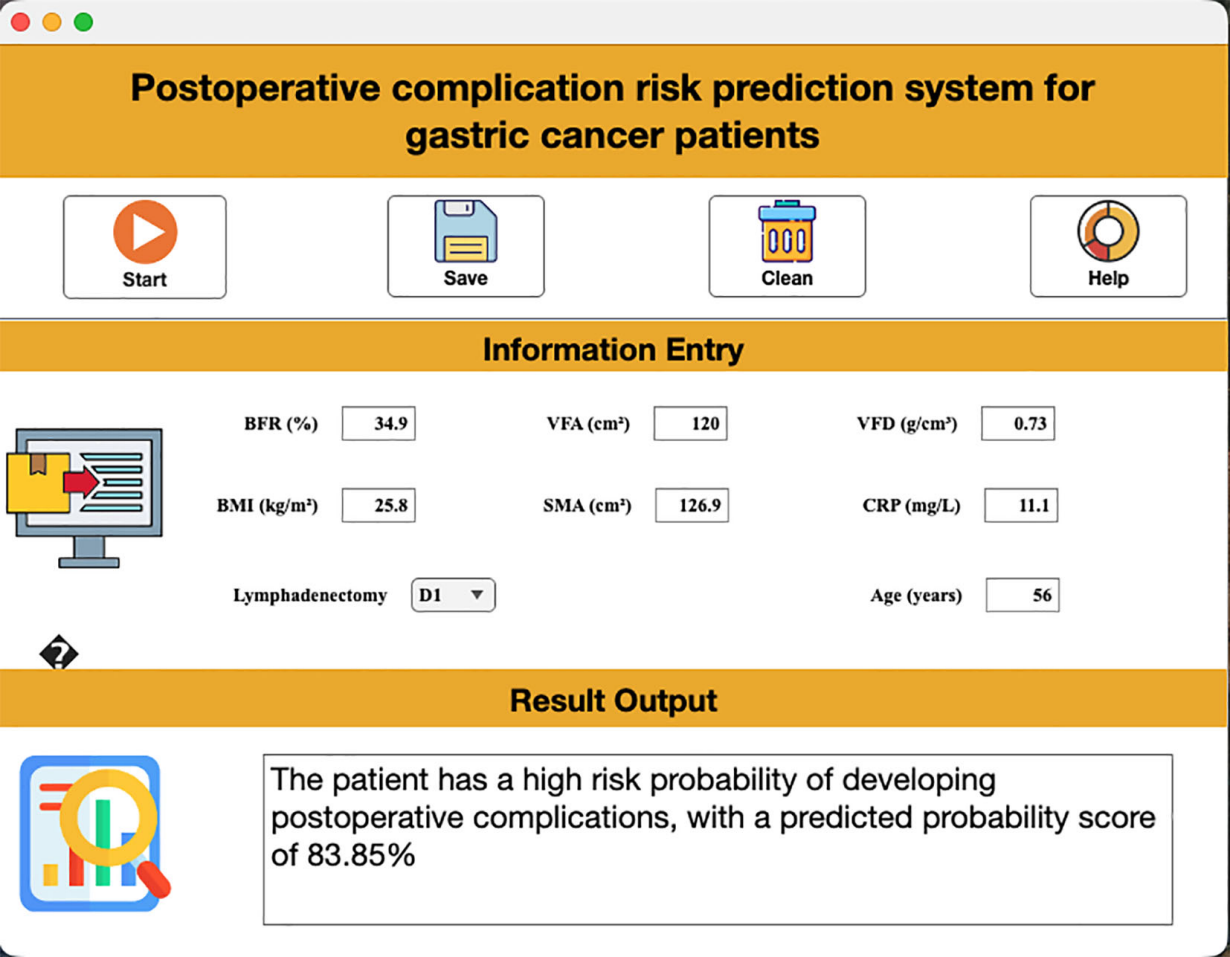
Visual interface demonstrating real-time risk assessment.

A proof-of-concept prototype visualization tool was developed that integrates the seven core predictors, providing support for analyzing the model’s decision logic in a research context. As demonstrated in **Figure 7**, clinicians input feature values on the dashboard to instantly compute postoperative complication risk.

## 4 Discussion

Gastric cancer incidence remains persistently high worldwide, with significant associations to obesity and metabolic diseases. Research indicates that higher BMI and visceral fat area (VFA) increase gastric cancer risk (10). Although treatment technology continues to advance, postoperative complications and tumor recurrence still pose severe threats to patient survival and prognosis (11). Consequently, identifying effective predictive indicators to improve surgical outcomes has become crucial.

The features selected were BFR, VFD, VFA, SMA, CRP, BMI, Age, lymphadenectomy extent. SHAP analysis showed identical importance ranking, confirming these as key predictors. This correlation occurs because SHAP values directly reflect features’ explanatory power: body composition metrics rank highest due to their strong nonlinear, highly specific relationship with prognosis. These indicators comprehensively reflect the body’s antitumor “baseline status,” providing critical predictive information (12). BFR had the highest mean absolute SHAP value, indicating its strongest independent contribution, and SHAP patterns are consistent with prior biological hypotheses proposing BFR as an integrated biomarker linking nutrition metabolism and immune function (13, 14). Beyond predictive superiority, BFR’s clinical implementation offers practical advantages: as a radiation-free assessment requiring only non-specialized equipment, it substantially reduces operational costs compared to CT-derived indices while capturing pathophysiological processes like sarcopenic obesity—patterns fundamentally invisible to BMI-based assessments. This positions BFR not merely as a supplementary metric, but as a paradigm-shifting biomarker that redefines perioperative risk evaluation frameworks. VFA and VFD influenced prognosis through direct mechanisms affecting tumor metastasis (15). SMA’s importance stems from its supportive role in bodily repair and antitumor immunity (16). CRP’s prognostic “warning function” amplified above 10 mg/L, but its independent SHAP contribution ranked lower than body composition metrics due to its dependence on metabolic status, though still higher than treatment-related indicators (17, 18). As a categorical variable, lymphadenectomy extent affects prognosis primarily through surgical standardization quality, with D2 dissection plus CRP>10 mg/L representing a high-risk combination requiring enhanced monitoring (19). BMI had the lowest SHAP importance because it cannot distinguish detrimental visceral obesity from protective muscle mass (20).

This study systematically evaluated six machine learning models for postoperative complication risk prediction in gastric cancer patients, demonstrating AutoML’s superior robustness in independent testing. The significantly enhanced predictive calibration performance of AutoML models relative to alternatives stems from their ability to integrate multidimensional indicators, including body composition parameters (BFR, VFA), inflammatory markers (CRP), clinical treatment variables (lymphadenectomy extent), and nutritional metrics (BMI), which encompass continuous and categorical data types with inherent inter-feature correlations (21, 22). Unlike traditional machine learning approaches like logistic regression and random forests, requiring manual feature selection, encoding, and dimensionality reduction—processes vulnerable to human errors causing omission of critical features or introduction of redundant information—AutoML employs automated feature engineering modules. These automatically identify distribution patterns across feature types, performing missing value imputation, outlier handling, feature interaction construction (e.g., BFR×CRP multiplicative terms), and high-dimensional feature reduction. This comprehensively captures nonlinear relationships within multi-source data, overcoming performance bottlenecks caused by imperfect feature engineering in conventional models (23, 24). Furthermore, AutoML enables unified modeling of multimodal data without manual format conversion, significantly improving adaptability to complex prognostic indicator systems (25). The integrated model search framework exhaustively explores preset model libraries (including logistic regression, random forests, XGBoost) while automatically evaluating generalization capabilities through cross-validation. Concurrently, intelligent algorithms such as Bayesian optimization and grid search efficiently optimize hyperparameters (learning rate, tree depth, regularization coefficients), rapidly identifying globally optimal parameter configurations within limited computational resources. This automated workflow substantially reduces technical barriers to model development, allowing rapid generation of clinically viable models without requiring specialized machine learning expertise, thereby meeting clinical demands for efficient predictive tool development (26–28). Despite the robust discriminative capability of the model, potential clinical harms associated with misclassification warrant careful deliberation. False-positive predictions may precipitate unnecessary surveillance imaging or invasive procedures, thereby elevating healthcare expenditures and radiation exposure risks. Conversely, false negatives could delay critical interventions for conditions including anastomotic leaks and peritoneal metastases, potentially exacerbating prognostic outcomes through postponed therapeutic measures. To quantitatively mitigate these risks, our decision curve analysis demonstrates how model-guided decision-making outperforms conventional clinical strategies, particularly reducing overtreatment at threshold probabilities exceeding 20%. We therefore emphasize that predictions should be interpreted solely as probabilistic clinical decision support tools, not as definitive diagnostic determinants, to safeguard patient safety during implementation. Implementation Pathway: The integrated clinical decision support tool developed using MATLAB App Designer provides an executable solution for routine perioperative risk stratification. This platform enables surgeons to input patient-specific metrics – including demographics, clinicopathological characteristics, and crucially, body composition parameters – to generate real-time, individualized complication probability estimates visualized through interpretable SHAP plots. For the CDSS interface, no formal usability testing, clinician feedback collection, or decision-impact analysis has been conducted in this research phase. Its purpose is solely to visually demonstrate interpretability mechanisms, not as a clinical deployment-ready system. To enhance accessibility and mitigate implementation barriers related to VFA/VFD quantification via specialist-dependent CT analyzes, our framework strategically incorporates BFR as a radiation-free, cost-effective surrogate biomarker validated through feature interaction analysis. Future deployment will leverage multicenter cohorts to refine data interoperability via blockchain-secured protocols, standardizing variable acquisition while minimizing heterogeneity linked to regional treatment variations. This dual approach positions the model for phased integration into preoperative assessment clinics, ultimately guiding nutritional interventions and surgical planning.

This study established a comprehensive research framework covering “data processing-model refinement-practical validation” through systematic approaches. Despite these achievements, the following limitations persist: (1) Data limitations: The single-center design inherently restricts external validity and introduces potential selection bias from institutional treatment protocols. Completeness was constrained by clinical practice variations — some medical institutions failed to comprehensively collect body composition indicators such as BFR and VFD due to diagnostic workflow disparities or inadequate documentation standards. This resulted in missing key features for certain samples. Although AutoML-based automated imputation strategies (including median imputation) were applied, potential impacts on the model’s precision in capturing data characteristics remain. Furthermore, regarding performance generalization to a third hospital setting, our external validation using Chuzhou First People’s Hospital data acknowledges significant barriers as hospitals with differing CT imaging protocols or alternative measurement methods (e.g., bioelectrical impedance vs. CT-based VFD) may exhibit substantial feature distribution shifts. On the other hand, the end points of this study were only limited to the prediction of postoperative complication risk, and the long-term outcomes were not further observed and analyzed. (2) Model limitations: The “black-box” nature of AutoML integrated modeling remains unresolved. While SHAP analysis quantifies feature importance (confirming BFR as a dominant predictor), it cannot visually reveal complex nonlinear interactions among features. Clinicians face high interpretation barriers to understanding the model’s decision logic, impeding clinical translation efficiency. (3) Implementation challenges: Core indicators like BFR and VFA/VFD require quantitative abdominal CT analysis—a non-routine practice in postoperative care that increases costs and patient burden, reducing clinical accessibility. Critically, if BFR distributions differ substantially across hospitals (e.g., due to population demographics or measurement protocols), the model’s dependency on this predictor may require retraining or adaptive recalibration via local data. Our median imputation method partially mitigates missing data but cannot resolve systemic protocol variations. Unaccounted confounders—including regional disparities in surgical quality (lymphadenectomy standards) and genetic heterogeneity— could degrade predictive efficacy in real-world applications. Future multi-center collaborations must establish standardized body composition assessment protocols using low-cost alternatives and explicitly test robustness against ±20-30% BFR distribution shifts through prospective validation. Future work must enhance model adaptability by incorporating broader clinical contextual variables. Future directions include: Optimizing data management through multicenter prospective cohort studies with large samples to establish unified data collection protocols, reducing heterogeneity. Blockchain technology and intelligent data entry systems will be introduced to minimize human error and improve accuracy. Concurrently, techniques like multiple imputation and Generative Adversarial Networks (GANs) will repair missing data to enhance dataset quality. Improving model performance entails developing low-cost alternatives for body composition assessment to reduce implementation barriers. Prospective clinical validation across healthcare tiers will refine generalizability in primary hospitals. Mobile or Hospital Information System (HIS)-integrated prediction tools enabling automated data import and real-time forecasting will be created to maximize clinical utility.

## 5 Summary

This research establishes an AutoML framework for predicting gastric cancer surgical outcomes, identifying eight core prognostic features and validating BFR as the dominant predictor through SHAP analysis. Though demonstrating excellent performance, clinical implementation requires overcoming data heterogeneity and model interpretability challenges. Future work will optimize practical application through multi-center collaboration and technical innovations.

## Data Availability

The raw data supporting the conclusions of this article will be made available by the authors, without undue reservation.
