# Supplementary material for: Development and clinical application of a postoperative complication prognosis prediction model for gastric cancer patients based on automated machine learning with body fat rate
Source: Front Oncol. 2026 Mar 3;16:1763139. doi: 10.3389/fonc.2026.1763139 (PMC12991994; doi:10.3389/fonc.2026.1763139)
Supplement: Supplementary file 1 [file DataSheet1.docx]

**Appendix A**

| PSEUDOCODE IHOA-AutoML Framework |
| --- |
| %% IHOA-AutoML Algorithm Framework  function [M_final, F_opt, Theta_opt, perf] = IHOA_AutoML(D_train, D_test)  %% Parameter Initialization  Initialize: N, T_max_feat, T_max_hyper, mu, beta, alpha_0, gamma, lambda  %% Stage 1: IHOA-based Feature Selection  % Chaotic initialization to generate feature mask matrices  Initialize feature selection population P_feat using Logistic chaotic map  for t = 1:T_max_feat  % Fitness evaluation  for i = 1:N  F_i = feature subset selected based on mask P_feat(i, :)  Train baseline model C_base on D_train(F_i)  fitness(i) = evaluate(C_base, D_val) - lambda * \|F_i\|  end    % IHOA position update (feature space)  Calculate guiding probabilities based on fitness  for i = 1:N  Select guiding individual m_g  alpha = alpha_0 * exp(-gamma * t / T_max_feat) % Dynamic step size  step = alpha ⊕ Levy(beta) % Lévy flight  P_feat(i, :) = P_feat(i, :) + step .* (m_g - P_feat(i, :))  Binarize the updated mask via Sigmoid function  end  end  F_best = optimal feature subset  %% Stage 2: IHOA-based Hyperparameter Tuning  % Chaotic initialization of hyperparameter population P_hyper (in normalized space [0,1]^h)  for t = 1:T_max_hyper  % Fitness evaluation  for i = 1:N  Theta_i = decode(P_hyper(i, :)) % Map to actual hyperparameter space  Train target model M_target on D_train(F_best) using Theta_i  perf(i) = evaluate(M_target, D_val)  end    % IHOA position update (hyperparameter space)  Execute IHOA update mechanism similar to Stage 1  Constrain positions within [0,1]^h  end  Theta_best = optimal hyperparameters  %% Final Model Construction  M_final = train final model (M_target, Theta_best, D_train(F_best))  perf = evaluate(M_final, D_test)  Return: M_final, F_best, Theta_best, perf  end |
