# Supplementary material for: Development and clinical application of a postoperative complication prognosis prediction model for gastric cancer patients based on automated machine learning with body fat rate
Source: Front Oncol. 2026 Mar 3;16:1763139. doi: 10.3389/fonc.2026.1763139 (PMC12991994; doi:10.3389/fonc.2026.1763139)
Supplement: Supplementary file 2 [file DataSheet2.docx]

**Appendix B**

**Performance Evaluation of Algorithm Improvement**

The enhanced IHOA was benchmarked against the original HOA, Genetic Algorithm (GA), and Whale Optimization Algorithm (WOA) using all 12 benchmark functions from the CEC2022 test suite. Parameters included: variable dimension = 10, population size = 30, maximum iterations = 500, with 30 independent runs for statistical reliability. Box plots visualizing optimization stability demonstrated IHOA’s superior performance over other algorithms in most test functions (Figure S1). Convergence curve analysis further confirmed that IHOA achieved faster convergence with the lowest risk of entrapment in local optima during iterations (Figure S2). These results validate the significant advantages of IHOA in global optimization capability and convergence efficiency.


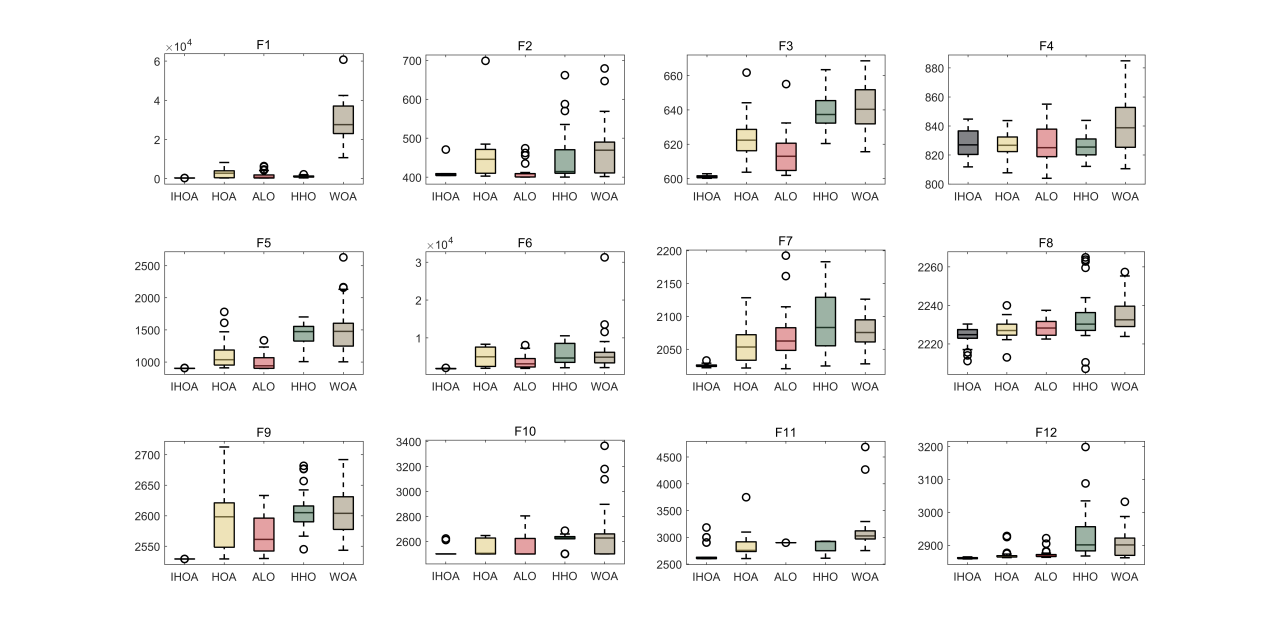


**Figure S1. Optimization performance comparison of swarm intelligence algorithms.**

**
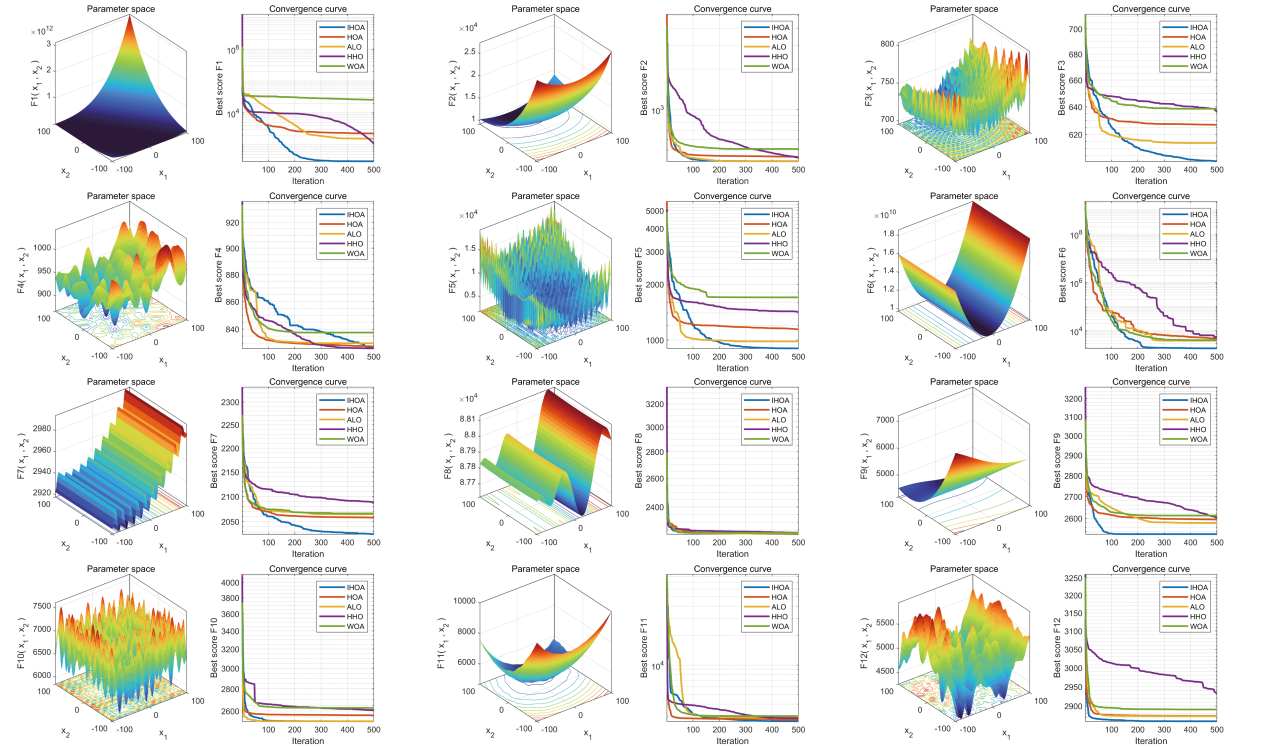
**

**Figure S2. Convergence performance comparison of swarm intelligence algorithms.**
